# Global Research Trends in Bladder Cancer Biomarkers: A Bibliometric Analysis

**DOI:** 10.7759/cureus.90724

**Published:** 2025-08-22

**Authors:** Alvin Tung Yong Zong, Wei Kang Yap

**Affiliations:** 1 Urology, Mersey and West Lancashire Teaching Hospitals NHS Trust, Liverpool, GBR; 2 Paediatrics, Newcastle upon Tyne Hospitals NHS Foundation Trust, Newcastle, GBR

**Keywords:** bibliometric analysis, biomarker, bladder cancer, research trends, urine

## Abstract

Bladder cancer has traditionally relied on cystoscopy, which is invasive and associated with patient morbidity, prompting extensive research into non-invasive biomarkers specific to bladder cancer. This study aims to map the global research trends in bladder cancer biomarkers over the last two decades using bibliometric analysis methodology. A total of 1337 articles from 2004 to 2024 were retrieved from Web of Science Core Collection and analysed using the Bibliometrix R Package (K-Synth Srl, Naples, Italy) and VOSviewer (Centre for Science and Technology Studies, Leiden University, The Netherlands). Research output in this field has grown from 21 publications in 2004 to a peak of 138 in 2021. China is the most productive country with 516 (38.6%) publications, and Sun Yat-sen University in Guangzhou, China, is the most productive institution. However, Spain, the United Kingdom, and Japan demonstrated greater research impact with higher average citations per paper. Shariat, SF, and Lotan, Y, are the two most influential authors in this field based on average citations and network analysis. Keywords analysis revealed a shift in research trends from established protein-based biomarkers such as NMP22 and UroVysion (Abbott Laboratories, Chicago, Illinois, United States) to novel genetic and epigenetic markers. Future studies should foster greater international collaboration in translating these novel biomarkers into routine clinical practice.

## Introduction and background

As of 2025, bladder cancer is the ninth most common malignancy in the world and the most common urinary tract cancer according to the International Agency for Research on Cancer (IARC) [1]. Currently, cystoscopy and direct visualisation of the bladder mucosa remain the gold standard for the diagnosis of bladder cancer. It is an invasive investigation associated with complications such as urinary tract infection, pain, and haematuria [2]. Additionally, repeated cystoscopies for cancer surveillance can increase patient anxiety and affect sexual performance [3]. Consequently, extensive research has been conducted into developing novel non-invasive urinary biomarkers for the diagnosis of bladder cancer.

At present, several of these protein-based biomarkers, including Nuclear Matrix Protein (NMP)22®, UroVysion™ (Abbott Laboratories, Chicago, Illinois, United States) using fluorescence in‑situ hybridisation (FISH), Bladder Tumour Antigen (BTA), and ImmunoCyt™ (DiagnoCure Inc., Québec, Canada) urinary biomarker, have been approved by the American Urology Association (AUA) in the United States as well as by the National Institute for Health and Care Excellence (NICE) in the United Kingdom [4,5]. Unfortunately, as many of these biomarkers lack the sensitivity and specificity of cystoscopy, they are currently only recommended as adjuncts to diagnosis and have yet to replace cystoscopy in clinical practice [6,7]. More recently, several new mRNA-based biomarkers have demonstrated promising results in diagnosing and monitoring bladder cancer. Biomarkers such as CxBladder (Pacific Edge Limited, Dunedin, New Zealand), ADX-Bladder™ (Arquer Diagnostics Limited, Sunderland, England), and Xpert Bladder® (Cepheid, Sunnyvale, California, United States) have demonstrated high levels of sensitivities and negative predictive values approaching those of cystoscopy in multiple prospective multicenter studies and could soon become an essential tool for bladder cancer diagnosis [8,9].

Objectives of the study

Despite significant recent breakthroughs in this field, at present, the global bibliometric landscape of this field remains largely unexplored. In this study, we used bibliometric analytical methods to determine the most influential papers and researchers in the field of bladder cancer biomarker research, understand the evolution of biomarkers over time, and identify current research gaps.

## Review

Methods

Articles were searched on the Web of Science Core Collection (WoSCC) on 4 February 2025, using the following search strategy: TI = (("biomarker*" OR "marker*" OR "non-invasive" OR "noninvasive" OR "non invasive" OR "NMP22" OR "Nuclear Matrix Protein 22" OR "BTA" OR "Bladder Tumor Antigen" OR "UroVysion" OR "Fluorescence In Situ Hybridization" OR "FISH" OR "Cxbladder" OR "Cxbladder test" OR "Cxbladder assay" OR "ImmunoCyt" OR "uCyt+" OR "ImmunoCyt/uCyt+ assay" OR "Xpert Bladder" OR "Xpert BC Monitor" OR "Xpert® Bladder Cancer Monitor" OR "UBC Rapid Test" OR "UBC®" OR "UroSEEK" OR "AssureMDx" OR "UroMark" OR "Bladder EpiCheck®" OR "EpiCheck" OR "ADX Bladder" OR "ADXBLADDER" OR "FGFR3" OR "Fibroblast Growth Factor Receptor 3" OR "TERT" OR "Telomerase Reverse Transcriptase" OR "microsatellite" OR "microsatellite instability" OR "liquid biopsy" OR "ctDNA" OR "circulating tumor DNA" OR "cell-free tumor DNA" OR "cfDNA" OR "cell-free DNA" OR "lncRNA" OR "Long Non-Coding RNA" OR "circRNA" OR "Circular RNA" OR "miRNA" OR "MicroRNA") AND ("bladder cancer" OR "bladder carcinoma" OR "bladder neoplasm*" OR "urothelial carcinoma" OR "urothelial cancer" OR "vesical cancer" OR "BC" OR "NMIBC" OR "MIBC" OR "urinary bladder cancer" OR "transitional cell carcinoma") AND ("diagnos*" OR "prognos*" OR "monitor*" OR "detect*" OR "FDA-approved" OR "commercially available" OR "clinical utility" OR "clinical validation" OR "emerging" OR "novel" OR "validation study" OR "sensitiv*” OR “specific*" OR "ROC curve" OR “ receiver operating characteristic*” OR "AUC" OR “area under curve”)) AND PY=(2004-2024) AND LA=(English).

Articles published between 2004 and 2024 and in the English language were selected. This time frame enabled analysis of early studies into bladder cancer biomarkers and explored the evolution of biomarker research over time. Only original articles and reviews were included in the analysis, while meeting abstracts, letters, editorial materials, and retracted publications were excluded from the records extracted. Articles retrieved were then meticulously screened by the two authors (ATYZ and WKY) independently for relevance based on title and abstract.

Data Collection and Analysis

**Figure 1:**
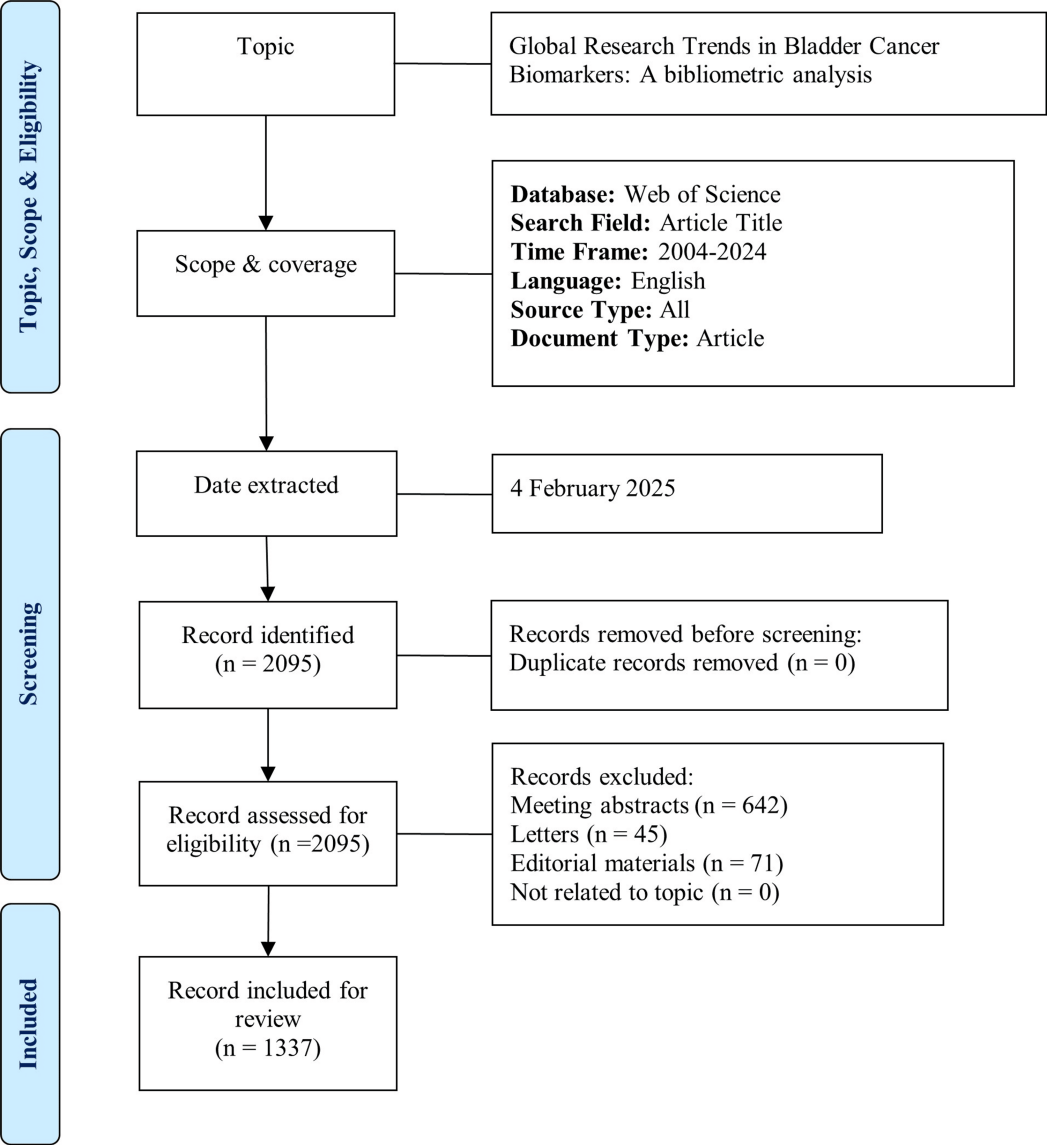
Flow diagram of the search strategy.

Figure 1 illustrates our search and selection strategy using an adapted Preferred Reporting Items for Systematic Reviews and Meta-Analyses (PRISMA) flowchart [10]. Initial data extraction was performed on 4 February 2025, which identified 2095 records. After screening, a total of 1337 articles were included in the final analysis. Bibliometric analysis and data visualisation were performed using VOSviewer version 1.6.20 (Centre for Science and Technology Studies, Leiden University, The Netherlands) and the Bibliometrix package (version 4.3.2; K-Synth Srl, Naples, Italy) based on the R language (version 4.4.2; R Foundation for Statistical Computing, Vienna, Austria, https://www.R-project.org/) and accessed through RStudio software. VOSviewer creates visual network maps by using the association strength method for normalization and a modularity-based algorithm for clustering, enabling visualization of relationships between keywords, authors, and institutions [11].

Results

Analysis of Publication Trends

**Figure 2:**
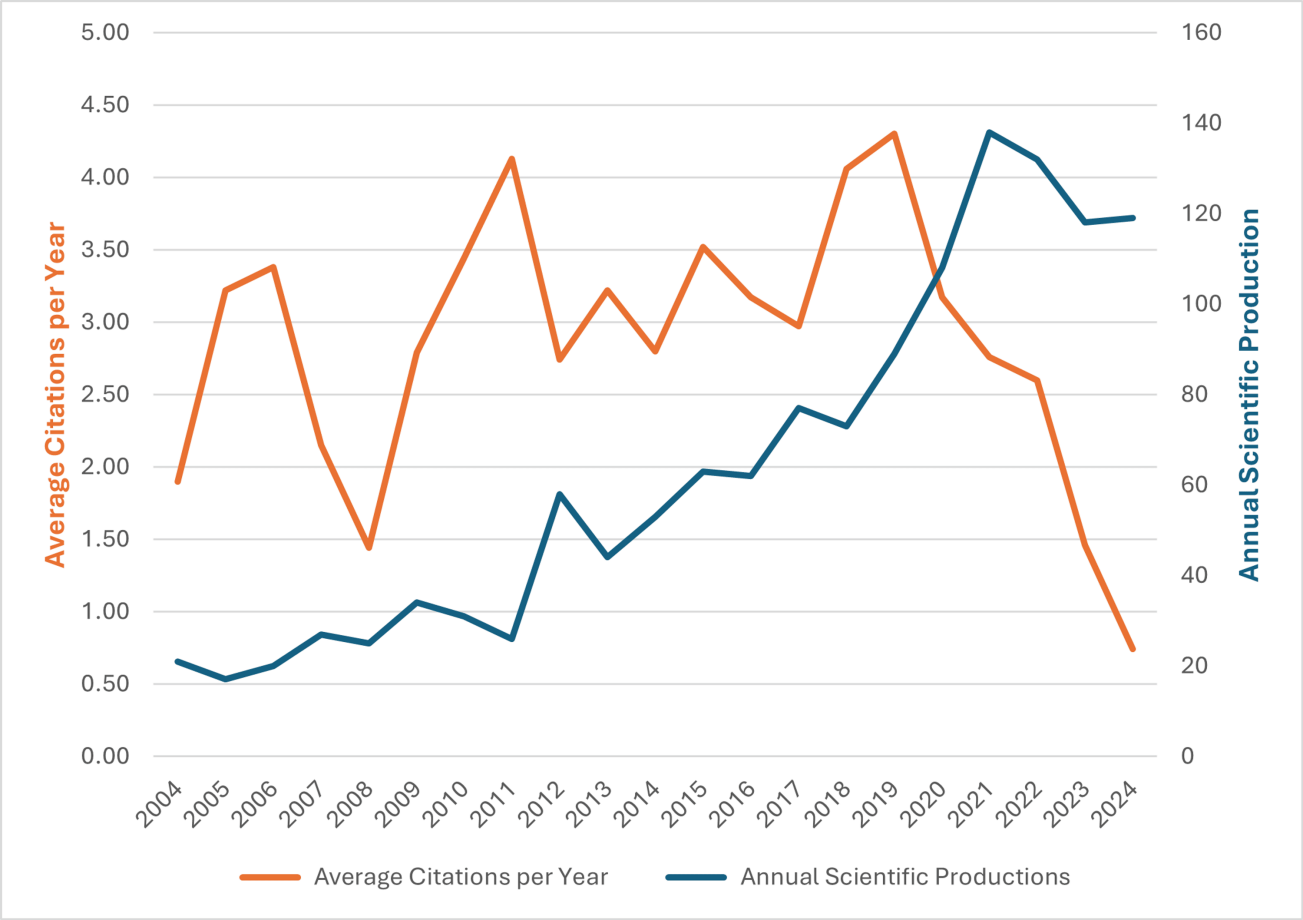
Annual scientific production and average article citation per year on bladder cancer biomarker research in 2004-2024.

Following the methodology of the PRISMA chart illustrated in Figure 1, a total of 1337 documents were identified, encompassing the work of 7430 authors from 1955 institutions across 47 countries, published in 368 distinct journals. As seen in Figure 2, the number of scientific publications related to biomarker research in bladder cancer has steadily increased from 21 in 2004 to 119 in 2024, peaking at 138 in 2021. The average number of citations per year remained relatively stable over the past two decades, with a peak of 4.30 in 2021.

Most Productive and Influential Countries

Table 1 presents the top 10 most productive and influential countries in this field. China produced the highest number of publications with 516 (38.6%) articles, followed by the United States (180, 13.5%), Japan (84, 6.3%), Germany (78, 5.8%), and South Korea (55, 4.1%). The top five countries with the highest total citations are China (9644), the United States (6366), Japan (3195), Germany (2455), and Spain (1482). In terms of average citations, however, Spain leads the way with 41.20 average citations, closely followed by the United Kingdom (38.40) and Japan (38.00).

**Table 1:** Top 10 most productive and influential countries in bladder cancer biomarker research (2004-2024).

Rank	Country	Publications, n (%)	Total citations	Average citations
1	China	516 (38.6%)	9644	18.80
2	United States	180 (13.5%)	6366	35.40
3	Japan	84 (6.3%)	3194	38.00
4	Germany	78 (5.8%)	2455	31.50
5	South Korea	55 (4.1%)	1180	21.50
6	Egypt	42 (3.1%)	775	18.50
7	Italy	39 (2.9%)	927	23.80
8	Spain	36 (2.7%)	1482	41.20
9	United Kingdom	30 (2.2%)	1151	38.40
10	India	27 (2%)	348	12.90

Most Active Journals

As seen in Table 2, the most active journal in this field is *Urologic Oncology: Seminars and Original Investigations*, which has published 58 articles, accounting for 4.34% of all publications in this domain. *Frontiers of Oncology* and the *International Journal of Molecular Sciences* are the next most prolific, with 46 (3.45%) and 33 (2.47%) publications, respectively. Other notable journals include *Cancers*, *Journal of Urology*, *Scientific Reports*, *European Urology*, and *Oncotarget*.

**Table 2:** Top 10 most active journals in bladder cancer biomarker research (2004-2024).

Rank	Journal Title	Publications, n (%)
1	Urologic Oncology: Seminars and Original Investigations	58 (4.34%)
2	Frontiers of Oncology	46 (3.45%)
3	International Journal of Molecular Sciences	33 (2.47%)
4	Cancers	31 (2.32%)
5	Journal of Urology	31 (2.32%)
6	Scientific Reports	28 (2.10%)
7	European Urology	27 (2.02%)
8	Oncotarget	27 (2.02%)
9	BJU International	23 (1.72%)
10	PLOS One	21 (1.57%)

Most Active Institutions and Authors

Chinese institutions dominate the list of most active research centers in this field (Table 3), making up nine out of the top 10 institutions. Sun Yat-sen University is the most prolific, with 58 publications (4.34%). It is followed by Peking University (46, 3.45%), Wuhan University (33, 2.47%), China Medical University (31, 2.32%), and Fudan University (31, 2.32%). The only non-Chinese institution in the top 10 is Chungbuk National University in South Korea, which ranks seventh with 27 (2.02%) publications.

**Table 3:** Top 10 most active institutions in bladder cancer biomarker research (2004-2024).

Rank	Institution	Country	Publications, n (%)
1	Sun Yat-Sen University	China	58 (4.34%)
2	Peking University	China	46 (3.45%)
3	Wuhan University	China	33 (2.47%)
4	China Medical University	China	31 (2.32%)
5	Fudan University	China	31 (2.32%)
6	Shanghai Jiao Tong University	China	28 (2.10%)
7	Chungbuk National University	South Korea	27 (2.02%)
8	Nanjing Medical University	China	27 (2.02%)
9	Chang Gung University	China	23 (1.72%)
10	Shandong University	China	21 (1.57%)

Table 4 summarises the top 10 most productive authors in the field of bladder cancer biomarker research. The most productive author is Wang, Y, with 43 (3.2%) publications and 1071 total citations. Following are Wang, J, with 40 (3.0%) publications, and Shariat, SF, with 35 (2.6%). In terms of average citations, Shariat, SF, and Lotan, Y, have the highest average citations, with 53.51 and 48.44 citations per paper, respectively.

**Table 4:** Top 10 most productive authors in bladder cancer biomarker research (2004-2024).

Rank	Author	Publications, n (%)	Total Citations	Average Citations
1	Wang, Y	43 (3.2%)	1071	24.91
2	Wang, J	40 (3.0%)	812	20.30
3	Shariat, SF	35 (2.6%)	1873	53.51
4	Lotan, Y	34 (2.5%)	1647	48.44
5	Zhang, X	33 (2.5%)	867	26.27
6	Li, H	32 (2.4%)	402	12.56
7	Wang, L	28 (2.1%)	1120	40.00
8	Wang, Z	28 (2.1%)	488	17.43
9	Li, J	26 (1.9%)	689	26.50
10	Zhang, Y	26 (1.9%)	683	26.27

Analysis of Highly Cited Documents

The most cited article in this field is "Rapid identification of UCA1 as a very sensitive and specific unique marker for human bladder carcinoma" by Wang et al., published in 2006, and has received 411 citations [12]. Other highly cited works include Ichimi et al.'s 2009 paper on microRNA signatures, which has 353 citations [13], and Christensen et al.'s 2019 study on cell-free DNA for detecting metastatic relapse, with 323 citations [14]. Table 5 summarises the top 10 most influential publications in bladder cancer biomarker research.

**Table 5:** Top 10 most influential publications in bladder cancer biomarker research (2004-2024). TC: total citations; FISH: fluorescence in situ hybridization

Rank	First Author	Title	Year	TC	TC/ Year
1	Wang, XS	Rapid identification of UCA1 as a very sensitive and specific unique marker for human bladder carcinoma [12]	2006	411	48.26
2	Ichimi, T	Identification of novel microRNA targets based on microRNA signatures in bladder cancer [13]	2009	353	9.43
3	Christensen, E	Early Detection of Metastatic Relapse and Monitoring of Therapeutic Efficacy by Ultra-Deep Sequencing of Plasma Cell-Free DNA in Patients With Urothelial Bladder Carcinoma [14]	2019	323	10.18
4	Malats, W	P53 as a prognostic marker for bladder cancer: a meta-analysis and review [15]	2005	248	6.11
5	Mowatt, G	Systematic review of the clinical effectiveness and cost-effectiveness of photodynamic diagnosis and urine biomarkers (FISH, ImmunoCyt, NMP22) and cytology for the detection and follow-up of bladder cancer [16]	2010	228	7.85
6	Allory, Y	Telomerase Reverse Transcriptase Promoter Mutations in Bladder Cancer: High Frequency Across Stages, Detection in Urine, and Lack of Association with Outcome [17]	2014	203	7.75
7	Zhang, DZ	Cell-Free Urinary MicroRNA-99a and MicroRNA-125b Are Diagnostic Markers for the Non-Invasive Screening of Bladder Cancer [18]	2014	200	6.14
8	Kamat, AM	ICUD-EAU International Consultation on Bladder Cancer 2012: Screening, Diagnosis, and Molecular Markers [19]	2013	195	12.83
9	Yamada, Y	Cell-Free Urinary MicroRNA-99a and MicroRNA-125b Are Diagnostic Markers for the Non-Invasive Screening of Bladder Cancer [20]	2011	182	4.56
10	Yu, J	A Novel Set of DNA Methylation Markers in Urine Sediments for Sensitive/Specific Detection of Bladder Cancer [21]	2007	179	5.14

Collaboration Network Analysis

**Figure 3:**
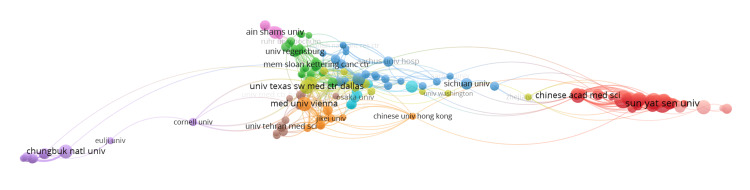
Institutional collaboration network.

Figure 3 highlights the institutional collaboration network in bladder cancer biomarker research. The size of the nodes corresponds to the number of publications, while the lines signify collaborative relationships between institutions. Chinese institutions (red cluster), led by Sun Yat-Sen University, form the largest and densest cluster in the network, with extensive collaboration seen within its own regional research ecosystem. The central cluster (blue, green, and yellow clusters) is formed by a mix of North American and European institutions, including Memorial Sloan Kettering Cancer Center in New York, United States, the University of Texas Southwestern Medical Center in Dallas, Texas, United States, and the University of Regensburg in Regensburg, Germany. A smaller, more isolated cluster (purple cluster) consists mainly of Korean institutions and is led by Chungbuk National University in Cheongju, South Korea.

**Figure 4:**
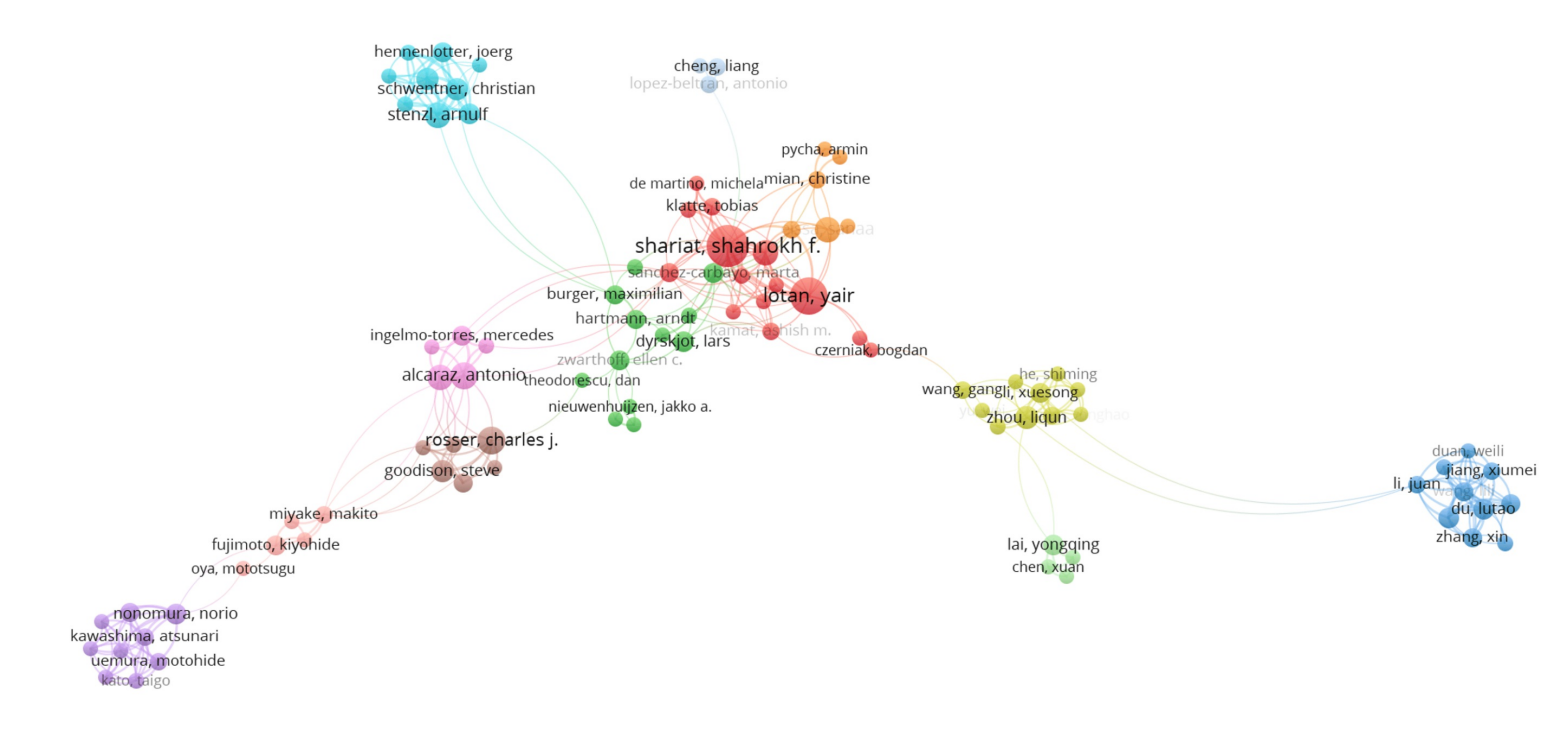
Author collaboration network.

As seen in the author collaboration network (Figure 4), Shariat, SF, and Lotan, Y are the two most productive and influential authors in this field of research, as evidenced by the size of their nodes and central position in the network. Two other major independent clusters can be seen in this analysis, comprising Chinese researchers (blue and yellow clusters) and Japanese researchers (purple cluster).

Keyword Analysis

**Figure 5:**
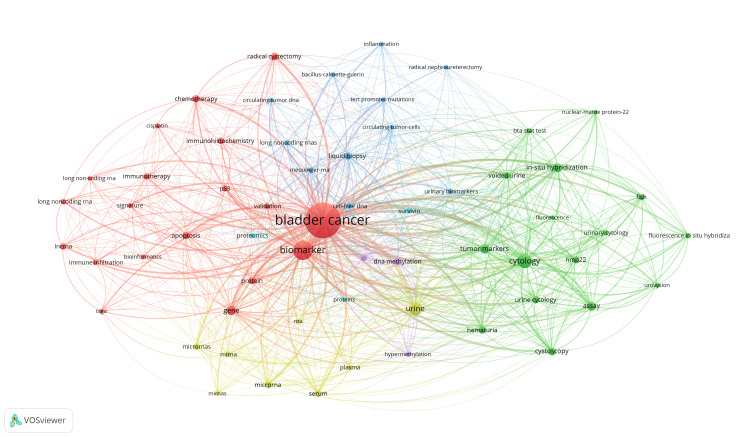
Keyword co-occurrence network.

There were a total of 4038 keywords, with 101 (2.50%) of them appearing 20 times or more. Figure 5 highlights the keyword co-occurrence network in this field and can be subdivided into four main clusters: red, blue, green, and yellow. The red cluster is the largest cluster and represents fundamental molecular entities such as “gene”, “protein”, and “long non-coding RNA” that form the foundation of biomarker research. The green cluster focuses on established diagnostic techniques such as “cytology”, “cystoscopy”, “nmp22”, and “urovysion”. The yellow cluster relates to microRNA changes in urine and serum plasma, with keywords such as “miRNA”, “urine”, and “serum”. Lastly, the blue cluster focuses on advanced genetic markers such as “TERT promoter mutation” and “circulating tumor DNA”.

**Figure 6:**
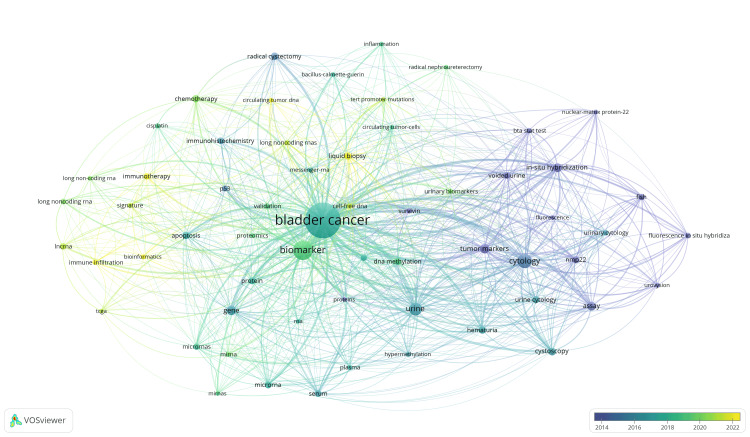
Keyword overlay analysis.

The overlay analysis seen in Figure 6 represents the temporal evolution of keyword usage in this field. Blue nodes represent older keywords (2014-2016) while yellow nodes represent emerging topics (2020-2022). Keywords such as “urovysion”, “cytology”, and “nmp22” were popular in the early 2010s and are now established diagnostic techniques in bladder cancer. In recent years, keywords such as “messenger RNA”, “long non-coding RNA”, and “circulating tumor DNA” have started trending and have become the new research focus in this field.

Discussion

**Figure 7:**
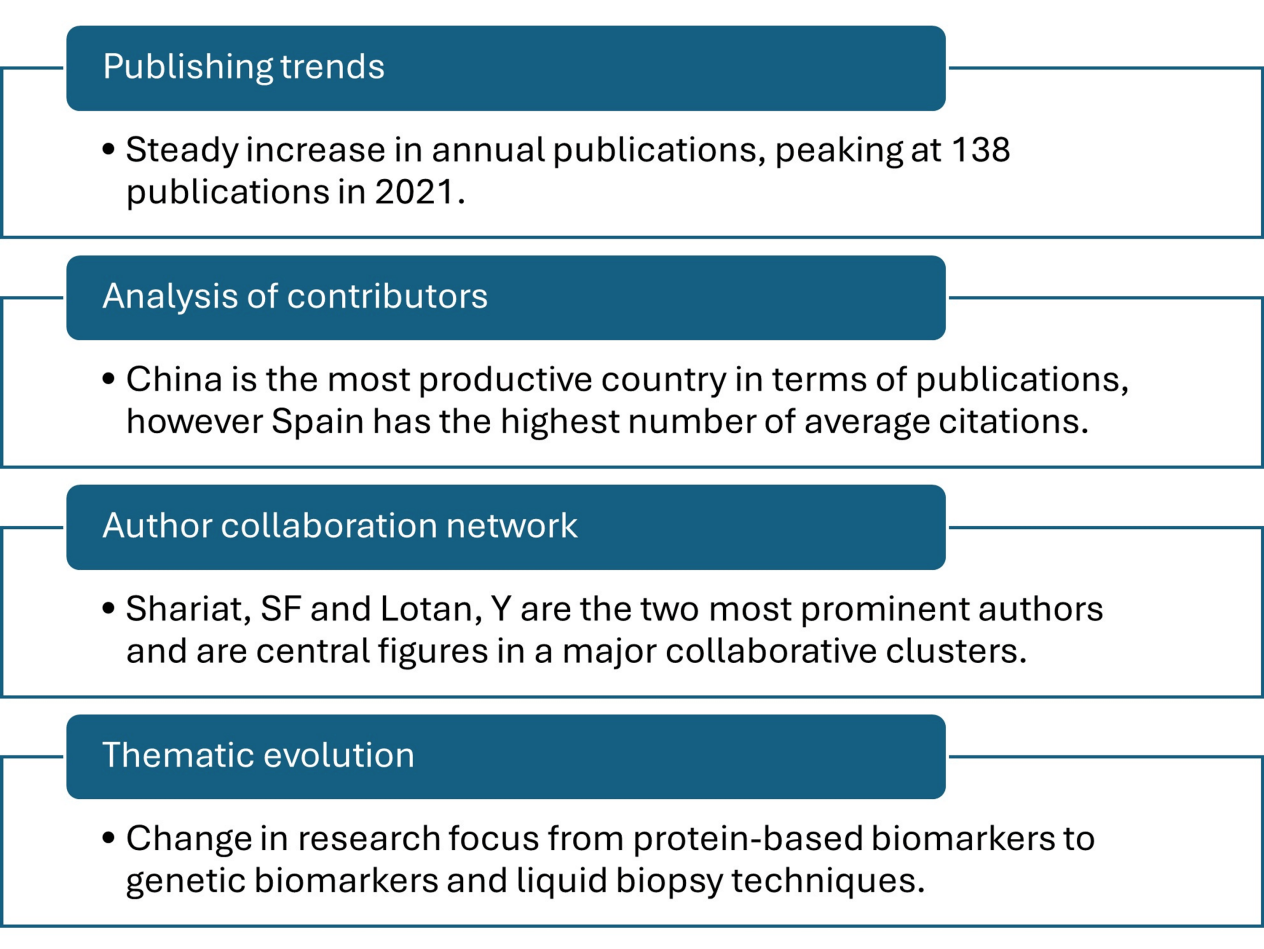
Key findings in bibliometric analysis of global research trends in bladder cancer biomarkers.

This bibliometric analysis provides a comprehensive overview of the global research landscape in bladder cancer biomarkers over the past two decades (2004-2024). Figure 7 depicts the key findings of this paper. The steady increase in annual publications, from 21 in 2004 to a peak of 138 in 2021, underscores the growing research interest and importance in this field. This trend reflects the ongoing clinical need for non-invasive diagnostic tools to replace or augment the current gold standard of cystoscopy, which is invasive and associated with patient morbidity. The sustained research output highlights a continuous effort to overcome the limitations of existing approved biomarkers, which often lack the required sensitivity and specificity to replace cystoscopy [22,23].

A key finding of this study is the significant contribution of Chinese institutions and authors to the volume of publications in this field. China is the most prolific country, producing 516 articles, with Chinese institutions making up nine of the top 10 most active research centers. Sun Yat-sen University is the leading institution with 58 (4.34%) publications. While China leads in the quantity of publications and total citations, countries like Spain, the United Kingdom, and Japan demonstrate a higher research impact, as indicated by their higher average citation rates. This suggests that while a large volume of research is emerging from China, studies from these other nations have been more influential in the field on a per-paper basis.

The most active journal in this field is *Urologic Oncology: Seminars and Original Investigations* (Impact factor (IF) = 2.3), which has published the highest number of articles on this topic. The presence of high-impact journals such as *European Urology* (IF = 25.2) and the *Journal of Urology *(IF = 6.8) in the top 10 list suggests that bladder cancer biomarker research is being published in leading urological journals, highlighting its significance in clinical practice.

The analysis of author and institutional collaboration networks reveals distinct patterns. The institutional network analysis shows a large, dense cluster of Chinese institutions with strong regional collaboration, and a separate, more isolated cluster of Korean institutions. A mixed cluster of North American and European institutions appears centrally, indicating established cross-continental collaborations. The author collaboration network further highlights the influential roles of Shariat, SF, and Lotan, Y, who are two of the most prominent authors in this major collaborative cluster. Shariat, SF, from the Medical University of Vienna, is the most influential author in this field, with an average citation of 53.51. He is closely followed by Lotan, Y, from the University of Texas Southwestern Medical Centre, who has an average citation of 48.44. Additionally, the presence of separate clusters of Chinese and Japanese researchers suggests that collaboration in this field may be geographically and linguistically isolated, particularly among East Asian researchers.

The thematic evolution of research focus in this field is clearly demonstrated by the keyword analysis. Earlier research, represented by keywords in 2014-2016, centered on established urinary biomarkers such as “nmp22”, “urovysion”, “bta stat test”, and “cytology”. These diagnostic tests have now received regulatory approval and are recommended in various national guidelines as adjuncts to cystoscopy [4,24]. In contrast, more recent keywords (2020-2022) such as “messenger RNA”, “circulating tumor DNA”, and “long non-coding RNA” signify a clear shift towards novel DNA and RNA-based diagnostics. Promising newer biomarkers, such as CxBladder and Xpert Bladder® detect various urinary mRNA (messenger RNA) signatures overexpressed in bladder cancer [25]. These tests have demonstrated significantly higher sensitivity and negative predictive value in detecting bladder cancer compared to older diagnostic tests such as urinary cytology and UroVysion™ [8,9]. This trend is further reflected by the most influential papers in this field, with the top 3 highest-cited publications exploring genetic and epigenetic biomarkers for bladder cancer [11-13]. More recently, “liquid biopsy” has emerged as a promising, non-invasive approach for the detection and surveillance of bladder cancer. By analysing biological fluids such as urine and blood, these tests identify tumor-derived material such as circulating tumor cells (CTCs), cell-free tumor DNA (ctDNA), and cell-free tumor RNA (ctRNA) [26]. Initial studies in this field are highly encouraging, suggesting this technology could play a pivotal role in replacing cystoscopy for bladder cancer diagnosis and monitoring [27].

Strengths and Limitations

This study has several strengths. This is the first bibliometric analysis, to our knowledge, to comprehensively map the global research landscape of bladder cancer biomarkers. The use of the Web of Science Core Collection and a timeframe of 2004-2024 ensures that an extensive list of high-quality literature is included in the analysis. However, the study is not without its limitations. The search was confined to articles in English, which may have led to the exclusion of relevant research published in other languages. Furthermore, by limiting the search to the article title, some important studies may have been omitted. The analysis also does not account for the varying clinical impact and real-world adoption rates of the biomarkers being studied.

## Conclusions

The field of bladder cancer biomarker research is a dynamic and rapidly expanding area of study. While China has emerged as a major contributor in terms of publication volume, influential research continues to be produced by established centres in North America and Europe. The research focus has markedly shifted from early-generation protein-based urinary biomarkers towards more complex genetic and epigenetic markers. Future research should focus on fostering greater international collaboration, particularly between Eastern and Western researchers, to translate these promising novel biomarkers into clinical practice, ultimately aiming to reduce the burden of invasive cystoscopic examinations for patients.
